# Freely Mobile Ocular Worm Hiding Away From the Light

**DOI:** 10.4269/ajtmh.21-0521

**Published:** 2021-07-12

**Authors:** Raghav Preetam, David Aggarwal, Brijesh Takkar

**Affiliations:** ^1^ The Cornea Institute, LV Prasad Eye Institute, Hyderabad, India;; ^2^ Srimati Kanuri Santamma Centre for Vitreoretinal diseases, LV Prasad Eye Institute, Hyderabad, India;; ^3^ Indian Health Outcomes, Public Health and Economics Research (IHOPE) Centre, LV Prasad Eye Institute, Hyderabad, India

A 65-year-old Indian man, a barber by profession, was referred for an intraocular foreign body noted after an uneventful cataract surgery 15 days prior. On examination, a long, slender, white structure was noted floating in the anterior chamber (Figure 1) that showed violent motion upon focus of light (Supplemental video). The patient was diagnosed to have a live intraocular worm, and its surgical extraction was immediately planned. However, the worm was no longer visible in the anterior chamber or seen on ocular sonography during preparation for surgery. Considering posterior migration of the worm behind the iris due to light exposure, vitrectomy surgery was planned. During surgery, intense exploration and globe indentation was performed, and the worm was finally located to be “hiding” in the anterior vitreous space behind the intraocular lens near the ciliary body (Supplemental video). Considering the chances of losing the worm from inside the eye due to its locale and its slender and mobile nature, in vivo lysis of the worm was performed uneventfully. The patient had a visual acuity of 20/30 with attached retina and no signs of ocular inflammation at 3 months of follow-up.

**Figure 1.**
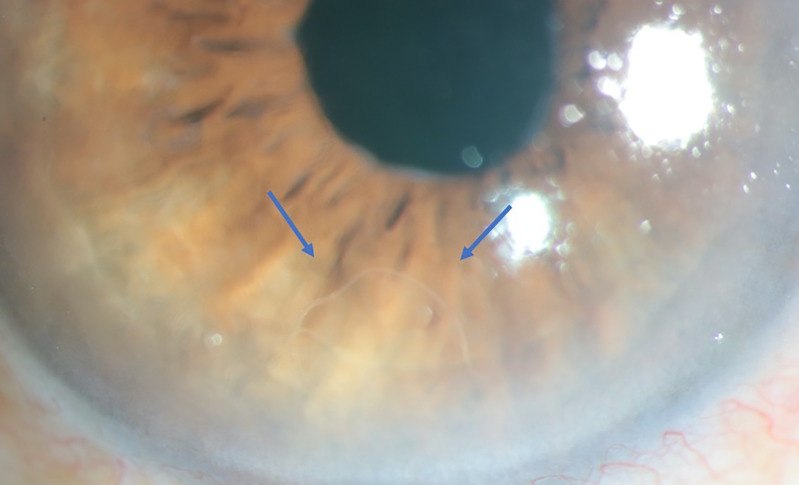
Still image shows the worm floating inside the anterior chamber (arrows). This figure appears in color at www.ajtmh.org.

Ocular parasitosis can have varied manifestations depending on the type of parasite.^1^ Because in vivo lysis was performed by us for the above-discussed reasons, the worm could not be identified or subjected to morphometric analysis. It was a thread-like worm with a round body and hence was likely a nematode. This appearance contrasted with that of a cysticercus (larval from of *Taenia solium*, a cestode known for ocular infection in tropical regions), which clinically appears as a whitish transparent to translucent cyst, often with a prominent scolex.^2^^,^^3^ Common human ocular infections by nematodes include angiostrongyliasis, filariasis (brugian and bancroftian), baylisascariasis, dirofilariasis, loiasis, onchocerciasis, thelaziasis, toxocariasis, and trichinosis.^1^ Considering the macroscopic features of the thin and white round worm that was hiding away from light and the endemic nature of infections in south India, dirofilariasis is the most likely infection in this case. The patient had no significant travel history. It is possible that the worm was already hiding behind the lens in the “dark vitreous cavity” before the cataract surgery was performed and was thus discovered later. Cataract surgery would have also allowed migration of the worm through the zonules. Iatrogenic transmission due to cataract surgery seems unlikely.

Regardless of presentation, the management of such cases should be urgent because the parasite can be mobile and may cause severe inflammation with vision-threatening sequalae.^2^ The violent reaction of the worm in response to focus of bright light was visible in the anterior segment as well as in the posterior segment (Supplemental video). In vivo lysis is a well-accepted way of managing such infections^3^ and proved very useful in our case of the migrating worm. In contrast to the ruffling motion associated with the scolex of the cysticercus larva,^2^ the worm seemed to be wriggling whenever the illuminator was focused on it, as if trying to “swim” away from the light (Supplemental video).

## Supplemental Material


Supplemental materials
